# Comparison of Emulsion Stabilizers: Application for the Enhancement of the Bioactivity of Lemongrass Essential Oil

**DOI:** 10.3390/polym16030415

**Published:** 2024-02-01

**Authors:** Lingling Liu, Kaleb D. Fisher, William D. Bussey

**Affiliations:** 1 Department of Agricultural and Biosystems Engineering, Iowa State University, Ames, IA 50010, USA; 2 Roy J. Carver Department of Biochemistry, Biophysics and Molecular Biology, Iowa State University, Ames, IA 50010, USA

**Keywords:** nanocellulose, surfactant, encapsulation, plant essential oil, control release

## Abstract

Recent focus on cellulose nanomaterials, particularly biodegradable and biocompatible cellulose nanocrystals (CNCs), has prompted their use as emulsion stabilizers. CNCs, when combined with salt, demonstrate enhanced emulsion stabilization. This study explored three emulsion stabilizers: Tween 80, soybean CNCs with salt (salted CNCs), and a combination of salted CNCs with Tween 80. Soybean CNCs, derived from soybean stover, were characterized by Fourier-transform infrared spectroscopy (FTIR), X-ray diffraction (XRD), differential scanning calorimetry (DSC) and thermogravimetric analysis (TGA). Antifungal testing against *Aspergillus flavus* revealed increased bioactivity in all lemongrass essential oil (EO)-loaded emulsions compared to pure essential oil. In addition, all three emulsions exhibited a slight reduction in antifungal activity after 30 days of room temperature storage. The release experiment revealed that the EO-loaded nanoemulsion exhibited a slow-release profile. The nanoemulsion stabilized by salted CNCs and Tween 80 exhibited significantly lower release rates when compared to the nanoemulsion stabilized solely by Tween 80, attributed to the gel network formed by salted CNCs. The findings of this study highlight the efficacy of cellulose nanocrystals procured from soybean byproducts in conjunction with synthetic surfactants to create nanoencapsulated essential oils, resulting in improved antimicrobial efficacy and the achievement of sustained release properties.

## 1. Introduction

Essential oils are plant secondary metabolites synthesized by plant cells with characteristics such as antimicrobial, antioxidant, anti-inflammatory, and anticancer activities [1]. Essential oils are normally present in plant storage structures, such as leaves, seeds, roots, barks, flowers, fruits, and rhizomes [2]. Essential oils are complex mixtures comprising over 300 compounds, predominantly falling within the terpenoid category, encompassing derivatives of esters, alcohols, phenols, along with a smaller fraction of phenylpropanoids [2]. Essential oils are highly volatile and sensitive to environmental factors including oxygen, light, and temperature [3], which limits their practical application. To enhance their stability and prolong their effectiveness, encapsulation techniques have been widely studied to enclose essential oils within protective substances [4].

Encapsulation of essential oils can be achieved mainly via capsules, hydrogels, emulsions, solid lipid nanoparticles, liposomes, and nanostructured lipid carriers [5]. It has been shown that encapsulated essential oils enhanced antimicrobial outcomes as compared to non-encapsulated counterparts [6]. In addition, nanoencapsulation, which encapsulates essential oils at a nanoscale, may exhibit better antimicrobial effects than its microscale counterparts due to an increase in specific surface area and more efficient interaction with microorganism cell membranes [7]. Essential oils can be nanoencapsulated by biopolymers such as nanocellulose [8], chitosan [9], and poly(ε-caprolactone) [10].

Oil-in-water emulsion is one of the most studied encapsulation techniques for essential oils. Oil-in-water emulsions can encapsulate lipid soluble components and enhance their bioaccessibility. Emulsions are thermodynamically unstable systems; thus, emulsifiers or emulsion stabilizers are normally added to enhance emulsion stability. Biopolymers and surfactants are widely used emulsion stabilizers. Cellulose nanocrystal (CNC) is one of the biopolymers that exhibits emulsion stabilization capability [11,12]. CNC stands out as a sustainable, organic, and environmentally friendly nanomaterial. CNC is usually prepared by acid hydrolysis of cellulose materials, with crystallinity index of 55–95% [13,14]. CNC exhibits dimensions ranging from 100 to 250 nm or extending to several microns in length, with a width spanning from 5 to 70 nm [15]. The average thickness of the interfacial layer of the CNC surrounding oil–water emulsion interface was reported to be 7–18 nm, depending on the charge of the CNC [16]. The emulsion stabilization capability of CNC was shown to improve upon the presence of salt [11]. In comparison to surfactants like Tween 80, biopolymers such as proteins and salted CNC (i.e., CNC in the presence of salt), exhibit lower surface activity and are less effective in reducing interfacial tension [17]. Nonetheless, emulsions stabilized with surfactants such as Tween 80 are vulnerable to environmental stresses [18]. The synergistic stabilization achieved through the combination of surfactants and biopolymers has been documented [8,17,19,20]. For instance, our previous study showed that the combination of salted CNC (i.e., CNC in the presence of NaCl) and Tween 80 could synergistically enhance the thermodynamic stability of nanoemulsions [20]. However, so far, very few studies have compared the bioactivity of essential oil-loaded emulsions stabilized by biopolymers and/or surfactants. Therefore, the antifungal properties of EO-loaded emulsions stabilized using salted soybean CNC, Tween 80, and a blend of salted CNC and Tween 80 were evaluated. CNC was derived from soybean stover, which comprises approximately 42% cellulose [21]. The chemical composition, thermal properties, and crystal structure of soybean CNC were thoroughly characterized. This study also explored the antifungal efficacy of an essential oil-loaded emulsion against *Aspergillus flavus*, a fungus known for causing significant food contamination and being linked to aflatoxin production [22]. The findings of this paper offer insights into the future development of essential oil-loaded emulsion products, as well as their potential applications in antimicrobial settings.

## 2. Materials and Methods

### 2.1. Materials

Lemongrass essential oil (*Cymbopogon Schoenanthus* Oil, reagent grade, 100%) was obtained from Spectrum Chemical MFG Corp (New Brunswick, NJ, USA). Tween 80 (P1754) was obtained from Sigma-Aldrich, Inc. (St. Louis, MO, USA). All other chemicals were acquired from VWR (Radnor, PA, USA) or Fisher Scientific (Pittsburgh, PA, USA). *Aspergillus flavus* Link 200026 NRRL 3357 strain was acquired from ATCC (Manassas, VA, USA). Soybean stover-derived CNC was made following our previously published method [20]. Firstly, soybean stover was collected from the farm, ground to below 1/8 of an inch, then washed for the removal of impurities. Following this, soybean stover was treated with an alkaline solution to mainly remove hemicellulose. After alkaline treatment, samples underwent a bleaching process to mainly eliminate lignin. Lastly, sulfuric acid hydrolysis and sonication were used on soybean stover to obtain soybean CNC. Soybean stover-derived CNC had a length of 117 ± 40 nm and width of 7.3 ± 2.0 nm as characterized by transmission electron microscopes (JEM-2100, JEOL Ltd., Akishima, Tokyo, Japan), with a mean particle size of 197 ± 6 nm, a polydispersity index of 0.65, and a zeta potential of −50 ± 1 mV as characterized by a Zetasizer Nano ZS instrument (Malvern Instruments Ltd., Worcestershire, UK).

### 2.2. Characterization of Soybean CNC

Soybean stover-derived CNC was lyophilized through the use of a freeze dryer (Labconco, Kansas City, MO, USA), then characterized by attenuated total reflectance Fourier-transform infrared spectroscopy (ATR-FTIR), X-ray diffraction (XRD), and differential scanning calorimetry (DSC)/thermogravimetric analyzer (TGA). Characterization using DSC/TGA was conducted with the STA 449 F1 Jupiter thermal analyzer (NETZSCH Instruments North America, LLC, Burlington, MA, USA). Samples were monitored over the temperature range of 33–800 °C with a constant heating rate of 10 °C/min under the flow of nitrogen gas with a flow rate of 20 mL/min to determine thermal stability. A derivative thermogravimetric (DTG) plot was made to display the mass rate of change of samples against temperature with respect to time.

The crystallinity analysis of soybean stover samples was evaluated by XRD utilizing an X-ray diffractometer (Ultima IV, Rigaku Americas Corporation, Woodlands, TX, USA) operating at 44 mA current and 40 kV voltage (wavelength = 1.54059 Å (Cu)) with a scanning rate of 2°/min from 10–50°. The crystallinity index value of each sample was calculated using the Segal method [23] depicted in Equation (1).

(1) $$CrI\;(\%)=\frac{I_{002}-I_{AM}}{I_{002}}\times 100$$

where CrI means the crystallinity index, $I_{002}\;$represents the maximum intensity of the (002) lattice diffraction (2θ around 22.6°), and $I_{AM}\;$refers to the minimum intensity of diffraction between (002) and (110) planes (2θ = 18°) accounting for amorphous scattering.

The FTIR spectra of samples were collected using an ATR-FTIR spectrometer (Bruker Tensor 37, Bruker Scientific LLC, Billerica, MA, USA) over a range of 4500~6000 cm^−1^ (4 cm^−1^ resolution, 32 scans/sample). Before measurement, the samples were combined with KBr (spectral grade) with a mass ratio of 1:99. The background spectra were collected, and baseline correction was performed for each sample spectrum.

### 2.3. Formulation and Characterization of Emulsion Stabilized by Soybean Nanocellulose and/or Tween 80

The as-prepared soybean CNC was used to encapsulate lemongrass essential oil (EO). According to our previous study [20], the encapsulation efficiency of soybean CNC was enhanced in the presence of salt (i.e., NaCl). Therefore, in this study, we prepared lemongrass essential oil (EO)-loaded emulsion stabilized by salted CNC and/or Tween 80. The emulsion samples were prepared according to the methods described in our previous publication [20]. Specifically, the nanoemulsion (labeled as EO + CNC + NaCl + T80) contained 5% EO, 1 wt% soybean CNC, 40 mM NaCl, and 10 wt% Tween 80. Several samples (labeled as EO + CNC + NaCl, EO + T80, and EO + water) were prepared as well and used as comparisons. Sample EO + CNC + NaCl refers to the emulsion formulated without Tween 80, while sample EO + T80 refers to the emulsion formulated without salted CNC. Sample EO + water refers to the emulsion formulated without Tween 80 and without salted CNC. All of the emulsion samples (10 mL for each) were subjected to ultrasonication using a 500 W ultrasonicator (Fisher Scientific, Hampton, NH, USA) at 60% amplitude (5 s on/2 s off) for 5 min. Some of the emulsion samples were tested fresh (labeled as fresh emulsion), while some emulsions were stored away from light, at room temperature, for 30 days before testing (labeled as 30-day old emulsion). Characterization of the emulsions including hydrodynamic particle size, zeta potential, and thermodynamic stability was shown in our previous publication [20]. In this study, further characterization of the emulsion droplet size was determined using a 200 kV JEM-2100 scanning/transmission electron microscope (STEM) (JEOL Ltd., Akishima, Tokyo, Japan).

### 2.4. Release Profile of Nanoencapsulated Essential Oil

The release profile of nanoencapsulated essential oil (i.e., EO-loaded emulsion stabilized by CNC and/or Tween 80 as shown in Section 2.3) was conducted according to Hou et al. [24] with modifications. Specifically, dialysis bags with a molecular weight cutoff from 12 to14 kD (Spectrum Chemical MFG Corp, New Brunswick, NJ, USA) were filled with nanoemulsion (10 mL) and dialyzed in 250 mL of deionized (DI) water with stirring. Aliquots (2 mL) of the dialysate were taken out at time intervals (2.5 min, 5 min, 10 min, 15 min, 20 min, 30 min, 1 h, 2 h, 3 h, 4 h, 5 h, 6 h, 23 h, 48 h, 72 h, and 7 days) with 2 mL of DI water being added back after collection. The absorbance of diluted dialyzed nanoemulsion in water was measured at 243 nm using a UV-Vis spectrophotometer (Azzota Scientific, Claymont, DE, USA). Using a standard curve of known essential oil concentrations (2–10 µg/mL) in 95% ethanol, the concentration of essential oil in the nanoemulsion was determined.

### 2.5. Antifungal Activity of Pure and Encapsulated Lemongrass Essential Oil

In vitro growth inhibition of *A. flavus* was performed using both pure and encapsulated essential oil (including fresh emulsion and 30-day-old emulsion) [25]. As the growth medium, sterile potato dextrose agar (PDA) containing 0.1% (*v*/*v*) Tween 80 was prepared. The parent emulsion (prepared as above) was diluted with PDA medium (at 55 °C) to reach certain EO concentrations (0.03%, 0.05%, 0.08%, 0.10%, and 0.15%). The resulting emulsion containing media were mixed well, then divided into Petri plates immediately. Plates were allowed to dry in the biosafety cabinet. Once plates were dried, 10 µL of 10^5^ conidia/mL spore suspension was applied to the agar surface for inoculation. The conidial suspension was prepared in sterile water and spores were scraped from two-week-old cultures. A hemocytometer was used for the counting. Following inoculation, the plates were then incubated for 14 days at 25 °C. The diameter of mycelial growth was recorded every 2 days over the 14-day time period. For each sample, four replicates were created and monitored. The antifungal activity of samples was measured using mycelium growth inhibition percentage (%MGI) as calculated below.

(2) $$MGI\left(\%\right)=\frac{d_{c}-d_{t}}{d_{c}}\times 100$$

where $d_{c}$ and $d_{t}$ refer to the diameter of mycelium in the control and treated plates, respectively.

### 2.6. Statistical Analysis

All measurements were conducted with at least triplicates. A significant analysis (α = 0.05) was conducted using ANOVA along with Duncan’s tests through SAS 9.4 statistical software (SAS Institute Inc., Cary, NC, USA).

## 3. Results and Discussion

### 3.1. FTIR Characterization

**Figure 1:**
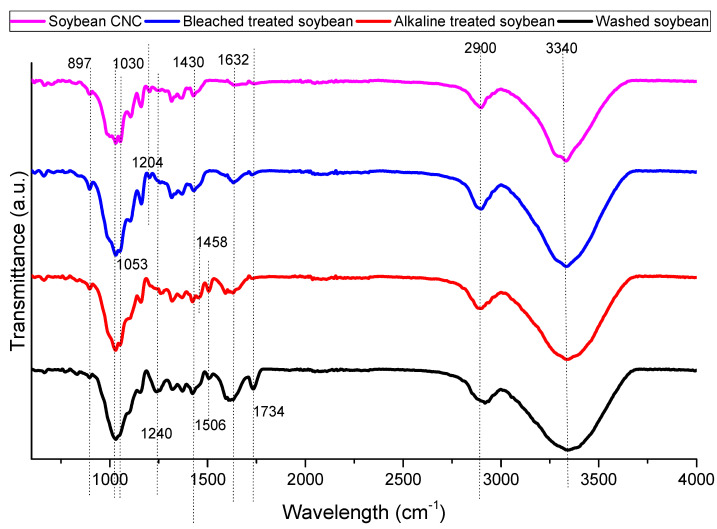
FTIR spectra of soybean stover samples following each treatment.

Figure 1 shows the FTIR spectrum of soybean stover samples after different treatments. All samples show some common bands around 897, 1030, 1430, 2900, and 3340 cm^−1^. The spectral bands at 897 and 1430 cm^−1^ are characteristics of cellulose I [26]. Specifically, the decrease in transmittance at 897 cm^−1^ represents the glycosidic C–O–C deformation of the β-glycosidic link in cellulose, while the spectral dip at 1430 cm^−1^ is representative of symmetrical CH_2_ bending. The band at 1030 cm^−1^ corresponds to the C–O and C–C stretching of cellulose [27]. The bands at 2900 and 3400 cm^−1^ are related to the C–H stretching vibration and aromatic O-H stretching vibration, respectively [28].

The band around 1060 cm^−1^ (1053 cm^−1^ in this study) is associated with cellulose. At 1053 cm^−1^, a more defined peak was measured in soybean CNC as compared to other samples indicating that its cellulose content was higher as compared to other samples [29]. At 1240 cm^−1^, CO-OR stretching in hemicellulose was observed, while the spectral band at 1734 cm^−1^ is representative of the C=O stretching of carbonyl and acetyl groups in the xylan components of hemicellulose [30]. The decrease in the intensity of both peaks following alkaline treatment indicates the elimination of hemicellulose from soybean stover.

The aromatic C=C stretching of lignin can be attributed to the band at 1458 cm^−1^ [28]. This band diminishes in bleach-treated soybean and soybean CNC as compared to alkaline-treated soybean indicating the removal of lignin. Also related to lignin is the band at 1506 cm^−1^ [30]. The disappearance of this band in bleach-treated soybean and soybean CNC as compared to washed soybean and alkaline-treated soybean also indicated that the bleaching treatment removed the lignin components in the soybean stover samples. The C=O stretching of the lateral chain of lignin may be related to the band at 1632 cm^−1^ [31]. The disappearance of this band in soybean CNC indicated its absence of lignin.

Both bleached soybean and soybean CNC had a peak at 1204 cm^−1^. The peak at 1204 cm^−1^ corresponds to S=O vibration in the sulfate ester groups in soybean CNC [29,30]. However, the removal of lignin was also reported to result in the appearance of new bands close to 1200 cm^−1^ due to the stretching of C-O bonds. For instance, bleached bagasse pulp had a new band at 1202 cm^−1^, while bleached wood pulp had a band at 1204 cm^−1^ [32].

### 3.2. XRD Characterization

**Figure 2:**
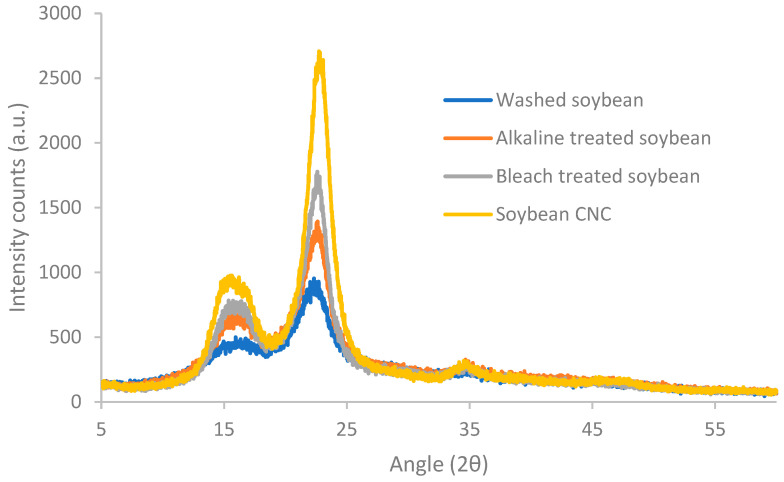
X-ray diffractograms of soybean stover samples following different treatments.

Diffraction patterns of soybean stover samples following different treatments are shown in Figure 2. After washing, soybean stover showed three reflection peaks at 2θ = 16.8°, 22.3° and 34.9°. These three peaks are typical cellulose type I crystals [33]. The peaks at 16.8°, 22.3°, and 34.9° correspond to the (101), (002), and (040) diffraction planes, respectively [33]. As shown in Table 1, soybean stover, after washing, displays a crystallinity index (59%) which is similar to that of soybean straw (56%) [34]. With consecutive treatments, the peaks in the XRD pattern appear narrower with higher intensity counts. Following the elimination of hemicellulose and lignin through alkaline and bleaching treatments, an increase in the crystallinity index to 69–73% was observed. A further increase in crystallinity values was observed after acid hydrolysis (82%), during which amorphous regions of cellulose were removed. Similar trends were observed by Neto et al. [35]. Specifically, the crystallinity index rose from 26% to 67% after alkaline and bleaching treatment, and the crystallinity index increased further to 73.5% following sulfuric acid hydrolysis [35].

The crystallinity of CNC can differ from 55% up to 95% [36], relying on the material sources and preparation conditions. The crystallinity index of soybean CNC (82% in this study) was much larger than that of soybean straw CNC (57%) [33], and soy hull CNC (73.5%) [35], but similar to that of CNC prepared from vine shoots (82% crystallinity) [30]. The higher crystallinity index of CNC observed in this study may be because a longer acid hydrolysis time (75 min) was incorporated while a shorter time (30–40 min) was applied in other studies [33,35]. Crystallinity index of nanocellulose (61.7–67.0%) [37] prepared from soybean straw with ball milling was also lower than that of soybean CNC prepared in this study.

### 3.3. DSC and TGA Characterization

**Figure 3:**
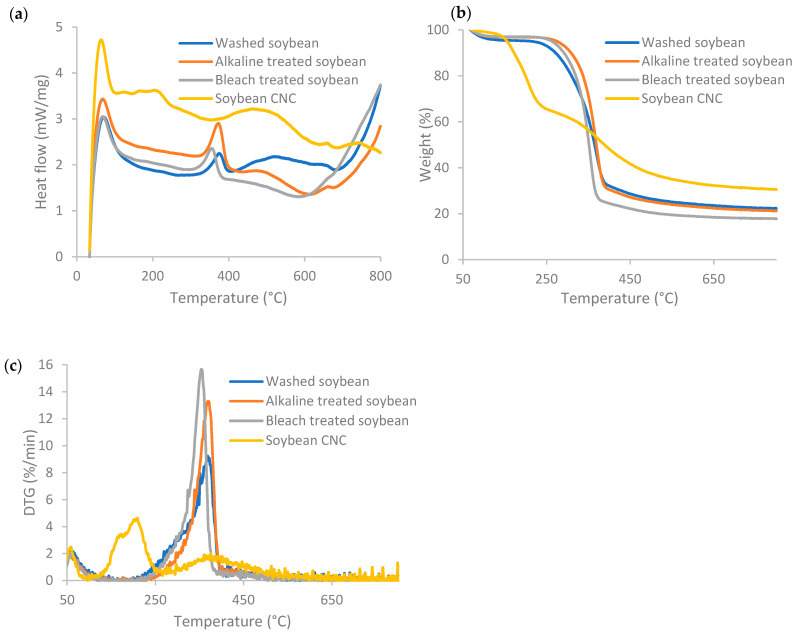
(**a**) DSC, (**b**) TGA, and (**c**) DTG profiles of soybean stover samples following each treatment.

Figure 3 displays the DSC, TG, and DTG profiles of soybean stover samples following each treatment. Weight loss at temperatures below 120 °C and an exothermal peak was seen for all samples. The weight lost below ~100 °C is caused by the evaporation of residual water in the samples, which is normally below 6% of the sample mass [38]. According to the TG curves in Figure 3b, the onset decomposition temperature for soybean CNC was found to be 160 °C, which is the lowest among all the samples. The onset decomposition temperature increased from 271 °C for washed soybean stover to 324–336 °C after alkaline and bleaching treatment. The sulfate ester groups present in soybean CNC led to a decrease at which thermal degradation took place, as the sulfate ester groups required less energy to become thermally degraded [26]. The diminished thermal stability of cellulose following introduction of sulfate groups was also reported in other studies [29,39].

Soybean CNC had a broad exothermal peak across a broad range of temperatures from 350 °C to 685 °C (Figure 3a). In general, the thermal degradation of cellulose includes dehydration, depolymerization, and breakdown of glycosyl units, and finally formation of a char residue [26]. For soybean CNC, thermal breakdown occurred in a broader range of temperatures and thermal degradation started at a lower temperature caused by the larger number of free ends in cellulose chains and its nano size [26].

The weight loss pattern of soybean CNC had two stages (Figure 3b), with one weight loss stage corresponding to the hydroxyl groups, and the other corresponding to the sulfate groups [38]. At lower temperatures, degradation occurred due to the presence of sulfated, amorphous regions, while the degradation of unsulfated crystalline regions was observed at the higher temperature stage [39]. This corresponds to the two peaks for soybean CNC at 207 °C and 375 °C in the DTG curves (Figure 3c). As a comparison, in the DTG curves, washed and alkaline-treated soybean stover had a peak at 369 °C, while bleach-treated soybean stover had a peak at 355 °C. The weight loss rate of the degradation process was the lowest in soybean CNC, as the increased sulfate contents resulted in lower degradation temperature and reduced maximum weight loss rate [39].

The residual weight of soybean CNC was 30.5%, which was the highest among all the samples. The residual weight of soybean stover slightly decreased after alkaline and bleaching treatments, from 22.2% for washed soybean to 21.1% and 17.8% for alkaline- and bleach-treated soybean, respectively. The increase in residual weight (or so-called char faction) of soybean CNC as compared to other soybean stover samples indicates that soybean CNCs can act as flame retardants [39].

### 3.4. Characterization of Nanoencapsulated Essential Oil

**Figure 4:**
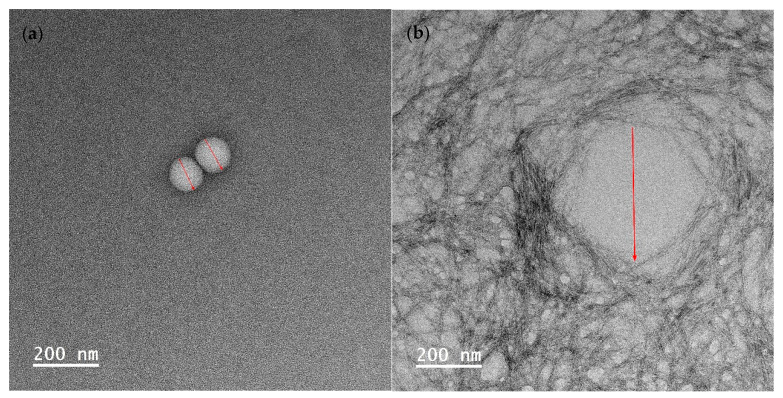

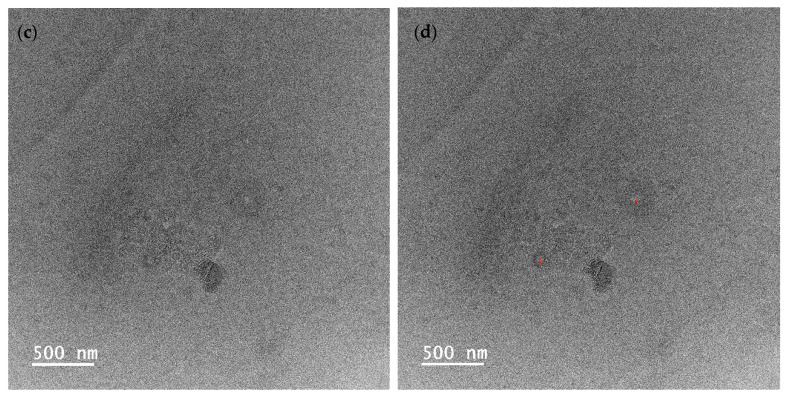
TEM images of encapsulated essential oil. (**a**) Essential oil encapsulated by tween 80; (**b**) essential oil encapsulated by soybean CNC in the presence of NaCl (i.e., salted CNC); (**c**,**d**) essential oil encapsulated by Tween 80 and salted CNC without and with marks. Note: The red marks represented the diameter of the emulsion droplets.

To visualize the droplet size of essential oil-loaded emulsion, TEM characterization was employed. As depicted in Figure 4, the average droplet sizes of emulsion (a), (b), and (c) were measured at 107 ± 4 nm, 394 ± 28 nm, and 60 ± 19 nm, respectively. This observation indicates that the emulsion droplet size is the smallest when stabilized by Tween 80 and salted CNC. In Figure 4b, it is evident that when stabilized solely by salted CNC, the oil droplets are surrounded by a salted CNC gel network. In the presence of Tween 80, the oil droplet is covered by Tween 80, and the salted CNC gel network further envelops the oil droplets. The TEM-characterized emulsion droplet sizes align with those determined by dynamic light scattering, as presented in our previous study [20]. Specifically, the average particle size of sample (a) was 49 nm, which was smaller than that of sample (b) (516 nm), and sample (c) (71 nm) [20]. The particle size distribution profile of the emulsion samples, as assessed through dynamic light scattering, is illustrated in Figure S1 and summarized in Table S1. The droplet size measured by TEM accurately represents the size of the emulsion droplets. In contrast, the particle size information obtained from dynamic light scattering comprises a composite of emulsion droplets, surfactants, and salted CNC.

### 3.5. Release Profile of Nanoencapsulated Essential Oil

**Figure 5:**
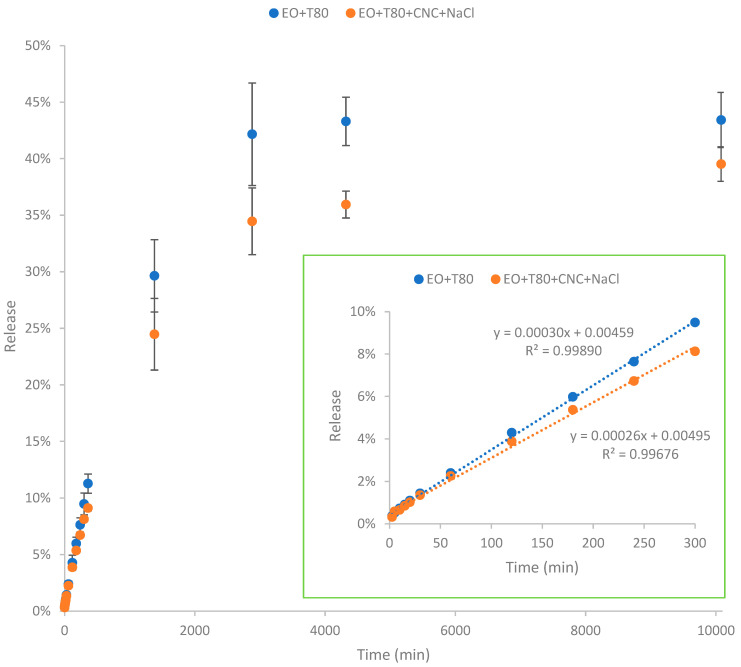
Release profile of nanoencapsulated essential oil over time. EO + T80 refers to encapsulation of essential oil by Tween 80; EO + T80 + CNC + NaCl refers to encapsulation of essential oil by Tween 80 and salted CNC.

According to Figure 5, essential oil in the nanoemulsion was gradually released from the nanoencapsulated systems. The release of EO reached almost steady after 7 days (i.e., 10,080 min). The nanoemulsion EO + T80 exhibited significantly higher release of EO compared to the nanoemulsion EO + T80 + CNC + NaCl. The total cumulative release of EO after 7 days for the nanoemulsion EO + T80 was 43.4%, which is higher than that (39.5%) of the nanoemulsion EO + T80 + CNC + NaCl. Because of the evaporation and degradation of essential oil as well as its low solubility in water, the overall released amount was lower than the initially input essential oil quantity (100%). In addition, the release rate was slowed down with the presence of salted CNC. Specifically, the initial release rate (corresponding to the slope of the graph) of the nanoemulsion EO + T80 was higher than that of the nanoemulsion EO + T80 + CNC + NaCl. The lower release rate indicates that the presence of salted CNC gel network can slow down the release profile of essential oils, restricting their diffusion. The significantly lower release rate of essential oils in the EO + T80 + CNC + NaCl sample may be mainly due to the high viscosity of salted CNC [40,41]. In this study, the diffusion profile of essential oil was similar to the diffusion of glucose in the presence of viscous nanocellulose as shown in our previous study [40,42]. Specifically, higher viscosity can result in lower diffusion rates [40,42]. 

Findings from this study are similar to those reported by Hou, Xu, Cen, Gao, Feng, and Tang [24]. Specifically, their study highlighted that cinnamon essential oil, when formulated in nanoemulsion stabilized by Tween 80 and hydroxypropyl-β-cyclodextrin (HPCD), exhibited a gradual release over a 400 min period [24]. The presence of HPCD slowed down the release rate of cinnamon essential oil, and higher HPCD concentrations resulted in lower release rates of cinnamon essential oil. This suggests that HPCD serves as an effective nanoencapsulation agent, facilitating sustained release of cinnamon essential oil and extending its release duration. Moreover, the study demonstrated that HPCD contributes to reducing the volatilization and environmental loss of cinnamon essential oil by providing cavity protection [43].

### 3.6. Inhibition of Mycelium Growth by Pure and Encapsulated Essential Oil

#### 3.6.1. Inhibition of Mycelium Growth by Fresh Pure and Encapsulated Essential Oil

**Figure 6:**
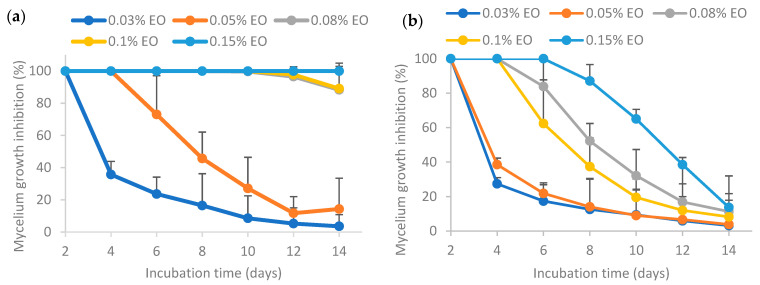

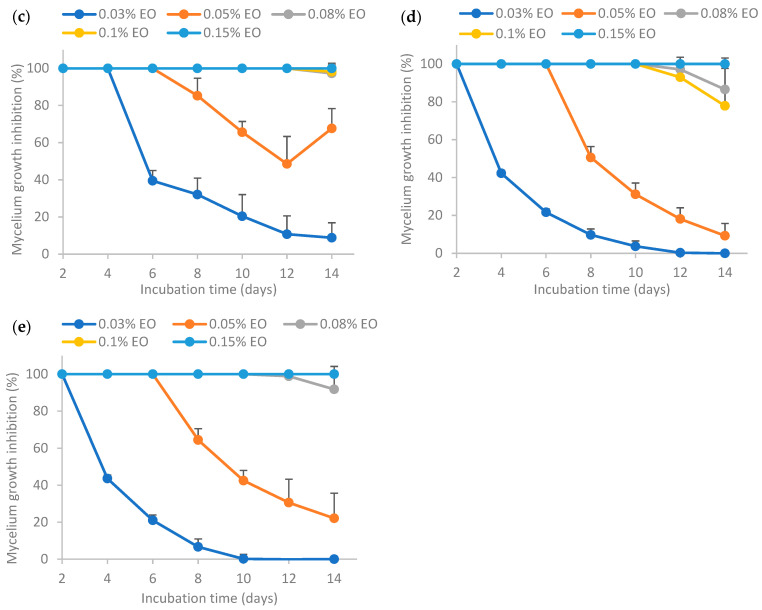
Effect of freshly prepared pure and encapsulated lemongrass essential oil on inhibiting the mycelium growth of *Aspergillus flavus.* (**a**) Pure essential oil; (**b**) essential oil in water; (**c**) essential oil encapsulated by Tween 80; (**d**) essential oil encapsulated by soybean CNC in the presence of NaCl (i.e., salted CNC); (**e**) essential oil encapsulated by Tween 80 and salted CNC.

Results from Figure 6 show that a higher EO concentration resulted in higher mycelium growth inhibition (MGI) rates. For pure EO (sample (a)), 100% inhibition of mycelium growth of *Aspergillus flavus* could be achieved at 0.15% EO or lower. In a nutshell, the MGI rates for the freshly prepared pure and encapsulated essential oil exhibited the following order: (c) >> (e) > (d) > (a) >> (b), where “>>“ indicates a significantly larger difference and “>“ denotes a non-significantly larger difference. EO encapsulated by Tween 80 (sample (c)) had the highest MGI rates, which were significantly different from other samples. EO encapsulated by Tween 80 and salted CNC (sample (e)) had significantly higher MGI rates than that of EO encapsulated solely by salted CNC (sample (d)). Both samples (d) and (e) had higher MGI rates than pure EO. Directly dispersed essential oil in water (sample (b)) without surfactants or emulsion stabilizers resulted in significantly lower MGI rates as compared to pure essential oil. This may be because lemongrass essential oil has low water solubility. These findings are similar to the antimicrobial activity reported for EO encapsulated by TEMPO-oxidized cellulose nanofibrils (TEMPO-CNF) and Tween 80 [8].

The higher antifungal activity of EO stabilized by Tween 80 (sample (c)) in comparison to Tween 80 and salted CNC (sample (e)) may be attributed to various factors. Firstly, the release of EO from sample (e) was observed to be significantly lower than that from sample (c) over time, as depicted in Figure 5. Secondly, the interfacial membrane surrounding the EO created a barrier that the EO had to traverse to interact with microbial cell membranes. As illustrated in Figure 4, the interfacial membrane of sample (e) was thicker than that of sample (c), suggesting that the efficacy of EO in sample (e) might be lower than that in sample (c). Nevertheless, the smaller emulsion droplet size in sample (e) could contribute to a more potent antimicrobial activity. In summary, the variations in the antimicrobial activity of EO stabilized by Tween 80 (sample (c)) or Tween 80 and salted CNC (sample (e)) result from these multiple factors.

The enhanced antifungal effectiveness of EO stabilized by Tween 80 (sample (c)) or Tween 80 and salted CNC (sample (e)) compared to EO stabilized solely by salted CNC (sample (d)) could be attributed to the smaller emulsion droplet size in the former cases, as indicated in Figure 4. Currently there are contradictory findings in the literature regarding the impact of emulsion droplet size on its antimicrobial effectiveness. Some studies report that the antimicrobial activity of essential oil-loaded emulsions was affected by droplet size, with nanoemulsion having higher antimicrobial activity than the coarse emulsion [44]. Conversely, other research suggests that antimicrobial oil-loaded emulsions with varied droplet sizes (i.e., micron-size and nano-size) had the same antimicrobial efficacy [45]. Nevertheless, Terjung et al. [46] demonstrated that essential oil-loaded nanoemulsion had reduced antimicrobial efficacy against *Listeria innocua* and *Escherichia coli C 600* as compared to macroemulsions ascribing to an elevated sequestration of antimicrobials at emulsion interfaces and a reduced solubilization within surplus Tween 80 micelles. Discrepancies in these findings may be attributed to variations in factors such as the type of antimicrobial oil tested, the specific microorganisms involved, and variations in emulsion formulation and preparation procedures.

Furthermore, conflicting findings also emerge regarding the mechanism underlying the antimicrobial activity of nanoemulsion. For instance, some studies reported that the remarkable antimicrobial activity of nanoemulsion was caused by their increased contact area with the microorganisms, thus an enhancement in the ability of bioactive compounds to penetrate cell membranes and increased accessibility of these compounds [47]. Conversely, other research contends that the antimicrobial efficacy of nanoemulsion can be attributed to the active ingredient instead of its high surface tension or cell wall diffusion activity [45]. These discrepancies may, in part, be attributed to variations in the antimicrobial agents and microorganisms tested, as well as differences in emulsion formulation and preparation procedures.

#### 3.6.2. Inhibition of Mycelium Growth by 30-Day-Old Encapsulated Essential Oil

**Figure 7:**
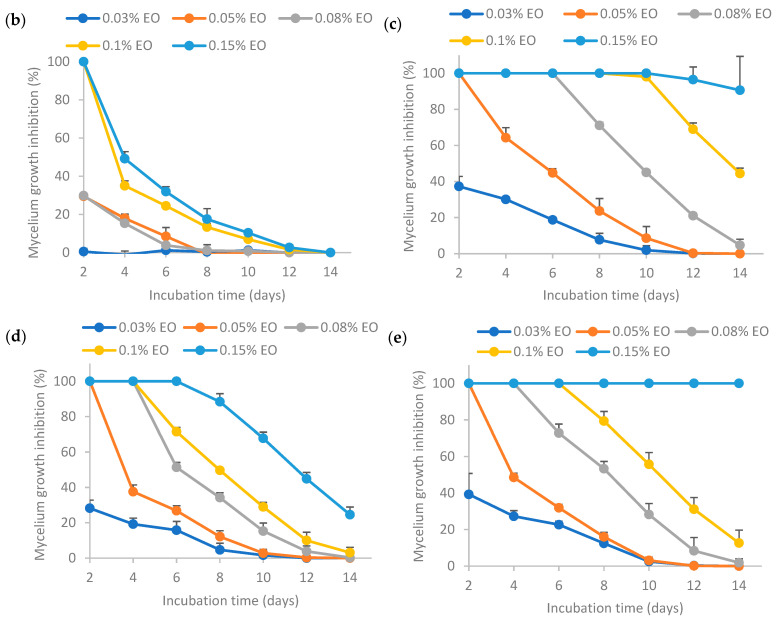
Effect of 30-day-old encapsulated lemongrass essential oil on the inhibition of *Aspergillus flavus* mycelium growth. (**b**) Essential oil in water; (**c**) essential oil encapsulated by Tween 80; (**d**) essential oil encapsulated by soybean CNC in the presence of NaCl (i.e., salted CNC); (**e**) essential oil encapsulated by Tween 80 and salted CNC. The samples were labeled to match the labeling in Figure 6.

The results from Figure 7 show that higher EO concentrations resulted in higher mycelium growth inhibition (MGI) rates. In a nutshell, the MGI rates for the encapsulated essential oil aged for 30 days adhered to the following order: (c) >> (e) >> (d) >> (b), where “>>“ signifies a significantly larger difference. This trend was similar to that of the fresh emulsion as shown in Figure 4, indicating that the storage time did not influence the relative antifungal activity among the samples. The MGI rate of EO encapsulated by Tween 80 (c) was significantly higher than others, indicating that the retention of EO activity was the highest when stabilized solely by Tween 80 after 30 days storage. The MGI rate of EO encapsulated by Tween 80 and salted CNC (e) ranked the second highest, which was higher than that of EO encapsulated by salted CNC (d). The MGI rate of EO in water (b) was the lowest, as sample (b) was not stable without the presence of emulsifiers or emulsion stabilizers.

Comparing the results between Figure 6 and Figure 7, it was found that the MGI rates decreased after 30 days’ room temperature storage for encapsulated essential oil. This may be because essential oil had low stability and high volatility [7]. Essential oil encapsulated by only Tween 80 (c) or by Tween 80 and salted CNC (e) could achieve ~100% MGI at 0.15% EO, indicating that these two emulsions were still effective at inhibiting *Aspergillus flavus* mycelium growth following a 30-day period of storage at room temperature. The formulation (c) and (e) had a better protection effect on EO than the other two formulations (essential oil in water (b) and essential oil encapsulated by salted CNC (d)).

**Figure 8:**
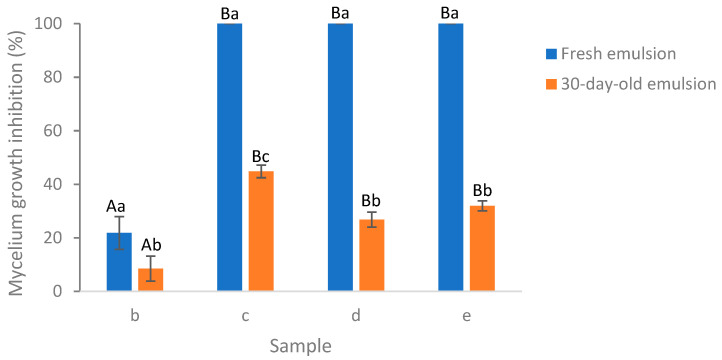
Comparison of fresh and 30-day-old emulsion containing lemongrass essential oil at 0.05% on the inhibition of *Aspergillus flavus* mycelium growth following a six-day incubation period on the plates. (**b**) Essential oil in water; (**c**) essential oil encapsulated by Tween 80; (**d**) essential oil encapsulated by soybean CNC in the presence of NaCl (i.e., salted CNC); (**e**) essential oil encapsulated by Tween 80 and salted CNC. The samples were labeled to match the labeling in Figure 6 and Figure 7. Samples labeled with uppercase letters showed significant differences (Duncan, *p* < 0.05) when compared across various emulsion samples, while samples marked with lowercase letters exhibited significant differences (Duncan, *p* < 0.05) when compared across different storage times.

Illustrating with the example of the encapsulated essential oil at 0.05% EO concentration, a comparison was conducted between the efficacy of the fresh emulsion and the 30-day-old emulsion in inhibiting *Aspergillus flavus* mycelium growth after a 6-day incubation of the plates (Figure 8). All the emulsion formulations showed a decrease in the MGI rates after 30 days room temperature storage. In particular, the MGI rates exhibited a significant decline, decreasing from 100% for the fresh emulsion to 45%, 27%, and 32% for emulsions (c), (d), and (e), respectively, which were 30 days old. Among the emulsions (b)–(e), the best retention of EO activity after 30 days storage was achieved in emulsion (c), which was significantly higher than that of emulsion (e), (d), and (a). The incorporation of salted CNC enhanced the retention of EO activity when comparing the emulsion (b) and (d). This may be due to the emulsion stabilization effect of salted CNC [11]. But maximum retention of EO activity was achieved in the presence of Tween 80 (c), which may be due to the emulsion stabilization effect of Tween 80. However, when comparing emulsions (c) and (e), the inclusion of salted CNC did not enhance the retention of EO encapsulated by Tween 80; instead, it led to a decrease. CNC suspension at 1 wt% exhibited no antimicrobial activity against *Aspergillus flavus* mycelium growth during the 14-day monitoring period (result not shown). The higher antifungal activity of emulsion (c) as compared to emulsion (d) and (e) may be due to the same reasons as described in Section 3.6.1.

## 4. Conclusions

In this study, CNC with a crystallinity index of 82% was prepared from soybean residues. It contained sulfate ester groups per FTIR analysis and exhibited a lower thermal degradation temperature than pretreated soybean stover samples. Soybean stover-derived CNC, in combination with Tween 80, serves as an effective stabilizer for lemongrass essential oil-loaded nanoemulsion. The nanoemulsion loaded with lemongrass essential oil displayed greater antifungal activity against the mycelium growth of *Aspergillus flavus* compared to pure essential oil. Moreover, the encapsulated nanoemulsion showed slight reduction in antimicrobial activity after room temperature storage for 30 days, with the nanoemulsion stabilized by Tween 80 exhibited the best retention of essential oil bioactivity. The nanoemulsion loaded with lemongrass essential oil displayed gradual release of essential oil. The inclusion of salted CNC (i.e., CNC in sodium chloride) slowed down essential oil release. These results suggest that soybean stover-derived nanocellulose in conjunction with surfactants holds promise for not only improving the antimicrobial properties of essential oils but also enhancing the thermodynamic stability of emulsions loaded with essential oils. This could potentially offer eco-friendly alternatives to chemical pesticides, in line with increasing demands for more natural products.

## Figures and Tables

**Table 1:** Crystallinity index of soybean stover samples after each treatment.

Sample	Crystallinity Index (%)
Washed soybean	59.27
Alkaline-treated soybean	69.29
Bleach-treated soybean	73.38
Soybean CNC	82.29

## Data Availability

The information provided in this research is accessible upon request from the corresponding author.

## Supplementary Materials

The following supporting information can be downloaded at: https://www.mdpi.com/article/10.3390/polym16030415/s1, Figure S1: Particle size distribution of emulsion samples encapsulated with lemongrass essential oil. (c) essential oil encapsulated by tween 80; (d) essential oil encapsulated by soybean CNC in the presence of NaCl (i.e., salted CNC); (e) essential oil encapsulated by tween 80 and salted CNC. The samples were labeled to match the labeling in Figure 6, Figure 7 and Figure 8. Table S1: Particle size information of emulsion samples encapsulated with lemongrass essential oil (corresponding to Figure S1).
