# Supplementary material for: Comparison of Emulsion Stabilizers: Application for the Enhancement of the Bioactivity of Lemongrass Essential Oil
Source: Polymers (Basel). 2024 Feb 1;16(3):415. doi: 10.3390/polym16030415 (PMC10857636; doi:10.3390/polym16030415)
Supplement: Supplementary file 1 [file polymers-16-00415-s001.zip › polymers-2750791-supplementary.pdf]

Supplementary Information

Comparison of emulsion stabilizers: application for enhancement of bioactivity of lemongrass essential oil

Lingling Liu [1,*], Kaleb D Fisher [2], William D Bussey [1]

[1] Department of Agricultural and Biosystems Engineering, Iowa State University, Ames, IA 50010, USA
[2] Roy J. Carver Department of Biochemistry, Biophysics and Molecular Biology, Iowa State University, Ames, IA 50010, USA
* Correspondence: Department of Agricultural and Biosystems Engineering, Iowa State University, Ames, IA 50010, USA; Telephone: (1)515-294-3939; E-mail: lingling@iastate.edu

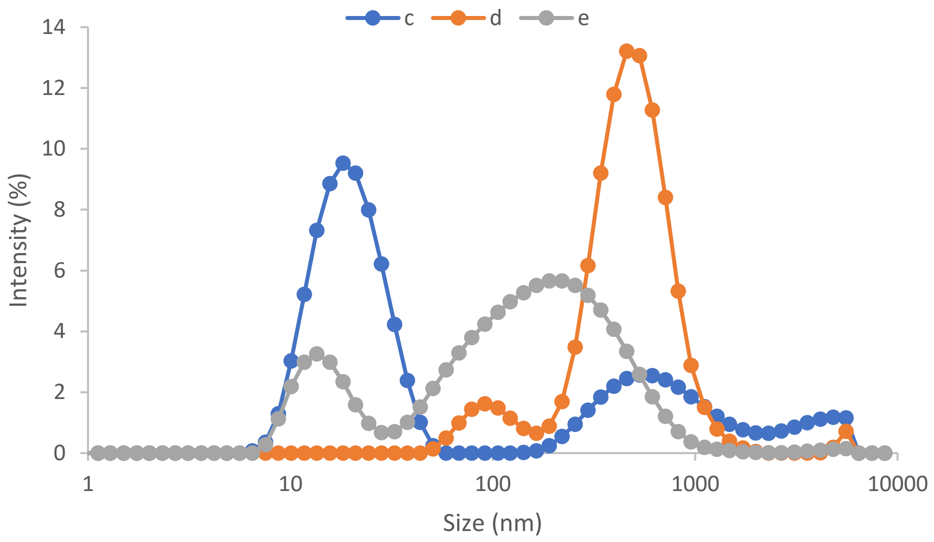

Figure S1 Particle size distribution of emulsion samples encapsulated with lemongrass essential oil. (c) essential oil encapsulated by tween 80; (d) essential oil encapsulated by soybean CNC in the presence of NaCl (i.e., salted CNC); (e) essential oil encapsulated by tween 80 and salted CNC. The samples were labeled to match the labeling in Figures 6 ~ 8.

Table S1 Particle size information of emulsion samples encapsulated with lemongrass essential oil (corresponding to Figure S1)

Sample	Peak 1[1] center (nm)	Peak 1 height (%)	Peak 2 center (nm)	Peak 2 height (%)	Peak 3 center (nm)	Peak 3 height (%)	Mean particle size (nm)	Median particle size (nm)
c	18.2	9.53	531	2.57	4800	1.18	49	22
d	91.3	1.62	459	13.21	5560	0.71	516	421
e	13.5	3.26	220	5.66	5560	0.15	71	132

[1]All the emulsion samples loaded with lemongrass essential oils had three peaks in the particle size distribution profiles. The center of the peaks (i.e., peak 1, 2, and 3) refers to the location of the peaks, while the peak height refers to the percent intensity at that peak.
